# Evaluation of β-Actin and Mitochondrial DNA Levels in Determining the Age of *Suidae* Remains

**DOI:** 10.3390/ijms252111674

**Published:** 2024-10-30

**Authors:** Krzesimir Szymankiewicz, Marek Walczak, Katarzyna Podgórska

**Affiliations:** Department of Swine Diseases, National Veterinary Research Institute, 57 Partyzantów Avenue, 24-100 Puławy, Poland; krzesimir.szymankiewicz@piwet.pulawy.pl (K.S.); katarzyna.podgorska@piwet.pulawy.pl (K.P.)

**Keywords:** β-actin, mtDNA, African swine fever, age, carcasses

## Abstract

African Swine Fever (ASF) is an infectious disease affecting pigs and wild boars, causing significant economic losses. Epidemiological surveillance plays an important role in minimizing its impact. The aim of this study was to evaluate the usefulness of β-actin DNA and mitochondrial DNA (mtDNA) levels in determining the age of animal remains from the Suidae family, which could be helpful in epidemiological investigations. The study included selected tissues and internal organs of Sus scrofa domesticus, exposed to natural environmental conditions or kept in stable 4 °C conditions, to assess the levels of β-actin and mtDNA over a period of 18 months. The levels of both tested parameters exhibited the expected decreasing trend over time. However, in most tissues, some discrepancies from this general pattern were observed. The results obtained for bone marrow showed a consistent downward trend and a strong correlation between mtDNA and β-actin, with long-term detectability (up to the 13th month of the study). Therefore, bone marrow could be considered a matrix of choice for age assessment. However, due to various influencing factors, further studies are required.

## 1. Introduction

ASF is a highly contagious viral disease affecting animals belonging to the Suidae family [1]. In Poland, ASF was first detected in 2014 [2]. Since then, the disease has been spreading steadily in the wild boar population, causing periodic outbreaks on pig farms. By the end of 2019, 25% of the country’s territory was covered by ASF risk zones [3]. Although the geographical expansion of the disease progresses slowly [4], human activity may speed up the spread of the ASF virus (ASFV) over long distances, as evidenced by the first introduction of ASFV into the Mazowieckie (2017) and Lubuskie (2019) voivodeships [5].

The detection of ASFV-positive wild boar carcasses in previously disease-free regions of Poland has created an urgent need to estimate the approximate age of these animal remains. This may be useful in determining whether ASFV had been present but undetected for an extended period in certain regions of Poland, or whether the ASF cases represent a completely new introduction of the disease. Several methods exist for determining the age of carcasses. Some are based on the estimation of changes that occur immediately after the animal’s death, such as the amount and distribution of rigor mortis [6]. It is also possible to estimate the age of a carcass based on its temperature [7] and hypostasis [8] or by determining the concentration of potassium in the vitreous humor [9]. However, the aforementioned methods are only effective for calculating the age of a carcass shortly after death (up to several dozen hours). For determining the age of carcasses over a longer period, important methods include evaluating the stage of decomposition and assessing the development and growth of insects present in the remains [10]. The adaptation of methods described in the forensic literature has proven helpful in estimating the time since death. The taphonomic model, often used to estimate the time of death of wild boar carcasses, was originally developed based on the decomposition of human bodies [11]. Rietz et al. proposed a model based on six stages of corpse decomposition, which allows for estimating the age of a corpse up to the fourth week [12]. While the aforementioned methods may be influenced by various factors, the aim of this study was to define easy-to-apply and measurable parameters useful for estimating carcass age. To achieve this, the stability (degradation over time) of two potentially suitable molecular parameters, namely β-actin- and mitochondrial-DNA (mtDNA) levels, was evaluated in an experimental study designed to address this question.

Scientific reports on the suitability of molecular biology techniques for estimating the age of a sample based on the quantitative assessment of its genetic material are limited. The few studies published in this field primarily describe the use of conventional PCR techniques for qualitative assessment, enabling approximate age estimation based on the presence of β-actin and mtDNA [13]. While β-actin content decreases with the aging of biological material [14], mtDNA exhibits greater stability and allows for the estimation of the age of samples older than six months [13]. In this study, we applied more sensitive real-time PCR to investigate the correlation between the age of biological samples and the levels of β-actin and mtDNA. For this purpose, selected fresh tissues and internal organs derived from a domestic pig (Sus scrofa domesticus) were exposed to natural environmental conditions over a period of 18 months to assess their impact on the rate of aging, measured by changes in β-actin and mtDNA levels monitored at one-month intervals.

## 2. Results

### 2.1. Stability of Outdoor Samples 

In contrast to the samples preserved at 4 °C, those stored outdoors exhibited noticeable changes in stability throughout the entire experimental period (0–18 months). As expected, the decomposition process advanced most rapidly in the kidneys and muscle tissue, which completely decomposed by the 6th and 7th months of the study, respectively. However, one kidney sample was found to be mummified, allowing for material collection up to the 12th month of the study. The last month of genetic material detection for each sample type is presented in Table 1.

### 2.2. Detection and Quantification of β-Actin

**Figure 1:**
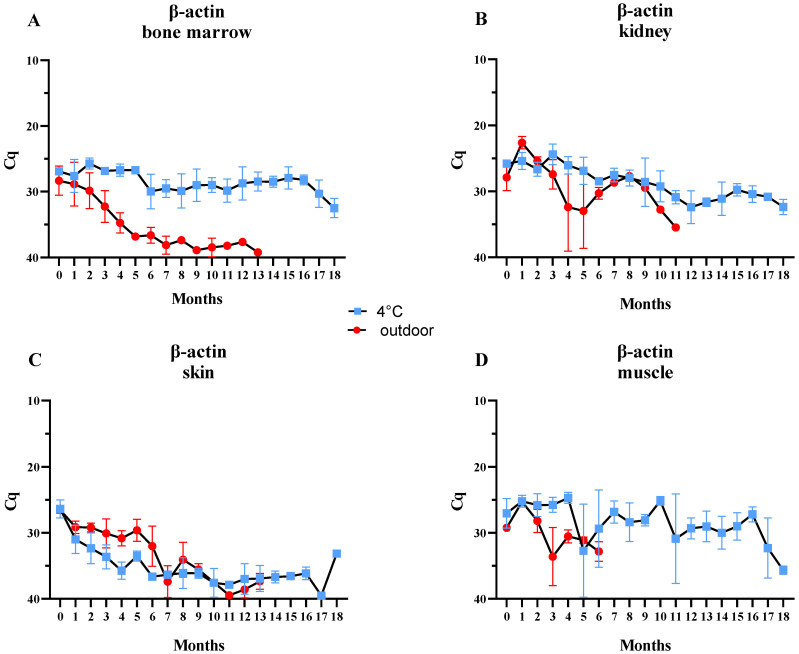
Average Cq values of β-actin for samples ((**A**) bone marrow, **B**) kidney, (**C**) skin, (**D**) muscle) stored in the refrigerator (blue points) and for samples located outdoors (red points) at monthly intervals. Error bars presenting standard deviation.

In the bone marrow, β-actin was detectable in triplicates until the 7th month, while qPCR-positive results were observed in one replicate up to the 13th month (Table 1). As shown in the graph (Figure 1A), the bone marrow exhibited a gradual decline in β-actin levels until the concentration of the genetic material fell below the detection limit. The exact increase in Cq between the first and last measurements was 10.91. As expected, the β-actin levels in samples stored at 4 °C remained more stable, decreasing more slowly compared to outdoor samples. Standard deviation (SD) values for both groups were similar (Figure 1A).

In the kidney, β-actin DNA detection was possible up to the 11th month in a single replicate (sampling up to 12th month), while in triplicates, it was detected only up to the 4th month (Table 1). The increase in Cq values over the eleven months was 7.57, although some fluctuations were observed. The β-actin levels in outdoor-kept samples displayed a downward trend during the first 5 months of the study, followed by a 3-month increase, and then a final decline toward the 11th month (Figure 1B). Similar to bone marrow, β-actin concentration in samples stored at 4 °C showed a more stable downward trend and lower standard deviation (SD) values compared to outdoor samples. 

The concentration of β-actin DNA extracted from skin samples exhibited a similar downward trend, regardless of storage conditions. Surprisingly, until the 10th month of the experiment, the outdoor samples had lower Cq values (indicating a higher concentration of DNA) than the samples stored at a stable 4 °C. Despite the noticeable downward trend over time, individual increases in β-actin concentration between the 5th and 13th months were evident (Figure 1C). β-actin in the skin samples kept outdoors was detectable for up to 13 months, and the total increase in Cq values during the experimental period (0–13 months) was 10.82.

The concentration of β-actin in muscle samples was unstable over time, exhibiting fluctuations and high standard deviations (Figure 1D). In the outdoor samples, β-actin was detectable until the 6th month of the experiment (with complete decomposition occurring by the 7th month), displaying a downward trend. Notably, there was an increase in Cq values in the 3rd month, with the difference between the first and last Cq values being 3.57 (Table 1).

In general, the β-actin plots, i.e., the graph lines representing the outdoor and fridge-kept samples, intersect multiple times for most of the examined sample types (kidney, skin, muscle) (Figure 1).

### 2.3. mtDNA

**Figure 2:**
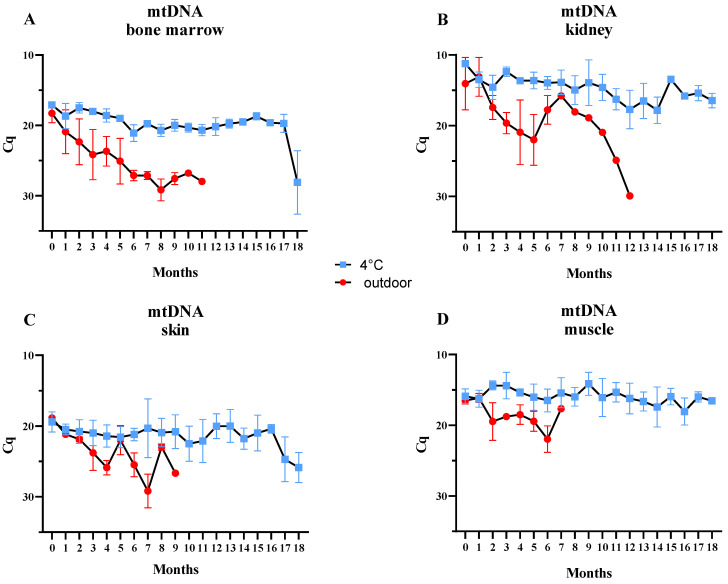
Average Cq values of mtDNA for samples ((**A**) bone marrow, (**B**) kidney, (**C**) skin, (**D**) muscle) stored in the refrigerator (blue points) and for samples located outdoors (red points) at monthly intervals. Error bars presenting standard deviation.

Similarly to β-actin, the mtDNA concentration in bone marrow samples proved to be much more stable at 4 °C compared to outdoor samples, where the mtDNA concentration exhibited a steady downward trend. MtDNA was detectable for over 11 months in outdoor-kept samples versus 17 months in fridge-preserved samples, with a rapid drop in mtDNA levels observed in the last month of the experiment (Figure 2A). In contrast to the β-actin assay, within-measurement standard deviations (SDs) were higher for outdoor-kept samples. The total increase in Cq value during the study period (0–11 months) was 9.68 (Table 1).

In kidney samples kept at 4 °C, the level of mtDNA remained relatively constant; however, high SD values were observed from the 8th to the 14th month, along with several slight increases in the 3rd and 15th months (Figure 2B). The Cq values of mtDNA extracted from kidney samples stored outdoors were unstable, showing an initial decrease in mtDNA levels from the 1st to the 5th month, followed by a subsequent increase over the next two months. From the 7th month onward, qPCR positive results were obtained for only one kidney sample, which demonstrated a continuous decrease in mtDNA levels up to the 12th month. The final increase in Cq value for the outdoor samples was 15.86 (Table 1).

In the case of skin samples kept at 4 °C, the Cq values of mtDNA were relatively stable until the 15th month; however, high SD values were noted between the 7th and 15th months (Figure 2C). In these samples, a sudden downward trend in mtDNA levels became apparent starting from the 16th month. For the outdoor samples, increases in Cq values were observed in the 5th and 8th months compared to preceding samplings, despite the overall downward trend. The difference in Cq values obtained between the first and last months was 7.81 (Table 1).

Among all tested samples, muscle tissue preserved at 4 °C exhibited the highest stability in mtDNA levels; the Cq values remained relatively consistent over the entire 18-month period (Figure 2D). In contrast, the mtDNA levels of samples stored outdoors displayed an initial downward trend, interrupted by slight fluctuations, ultimately indicating a concentration almost equivalent to that at the beginning of the experiment. Consequently, the difference in Cq values was only 1.16 (Table 1).

### 2.4. Correlation Between β-Actin and mtDNA 

The correlation between β-actin and mtDNA was assessed separately for the fridge-preserved and outdoor-kept samples. The calculation was based on the Pearson correlation coefficient (r) after an initial examination of normal distribution using the Shapiro–Wilk test.

**Figure 3:**
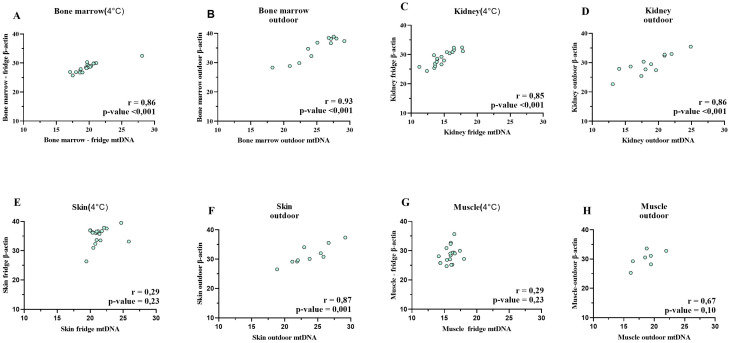
Correlation of β-actin and mtDNA in individual organs ((**A**,**B**) bone marrow, (**C**,**D**) kidney, (**E**,**F**) skin, (**G**,**H**) muscle) depending on the location of the samples.

In the case of bone marrow, a high positive correlation was observed for both storage conditions: r = 0.86 for fridge-preserved samples and r = 0.93 for outdoor samples (Figure 3A, Figure 3B). These results were statistically significant (*p* < 0.001).

A similar pattern was noted in the kidney samples, where the correlation coefficients were also high: r = 0.85 for samples at 4 °C and r = 0.86 for outdoor samples, with statistical significance (*p* < 0.001) (Figure 3C, Figure 3D).

In contrast, results for skin samples were different. In samples preserved at 4 °C, a low and statistically insignificant correlation was observed (r = 0.29, *p* = 0.23) (Figure 3E). Conversely, the correlation of β-actin and mtDNA in outdoor samples was high and statistically significant (r = 0.87, *p* = 0.001) (Figure 3F).

Similar discrepancies were observed in muscle tissue, where the correlation between tested parameters was r = 0.29, *p* = 0.23 for fridge-kept samples, and r = 0.67, *p* = 0.1 for outdoor samples (Figure 3G, Figure 3H).

## 3. Discussion

African swine fever is considered one of the most serious threats to the pig production sector with significant far-reaching consequences for the global economy [15]. Due to the severe progression of the disease in domestic pigs, the occurrence of ASFV infections in farm animals also raises significant animal welfare concerns. Wild boars, as natural hosts and long-term reservoirs of the virus, play a crucial role in the epidemiology of ASF [16]. Therefore, establishing an effective disease surveillance scheme that includes continuous monitoring of ASFV infections among susceptible animals and virus reservoirs in wildlife is a crucial component of the global ASF control strategy. As suggested by Palencia et al. [17], the effectiveness of implemented interventions against ASF in reservoir animals (e.g., carcass search, culling, fencing) should be evaluated based on the ongoing monitoring of the virus’s spatiotemporal spread and simultaneous tracking of changes in wild boar populations as an integrated approach. To date, managing the wild boar population and testing carcasses for the presence of ASFV are considered the most effective methods for combating early ASF in wild boars [18]. 

Nevertheless, none of the currently proposed control strategies is sufficient to completely interrupt the further expansion of ASFV. Therefore, there is an urgent need for new innovative and more effective tools facilitating molecular surveillance of the disease.

Scientific evidence indicates that ASFV can persist in decaying wild boar carcasses, posing a potential threat to further disease spread [17]. Although the virus can be detected in decaying carcasses for up to 35 days post-mortem under appropriate environmental conditions, the risk of a carcass becoming a source of infection for other susceptible Suidae species decreases over time following the animal’s death [19]. Nevertheless, the exact time frame during which the carcass of an ASFV-infected animal may serve as a reservoir for the infectious virus in the environment remains unknown. In addition to a detailed assessment of ASFV infectivity dynamics over time, determining the approximate age of wild boar carcasses could help address this knowledge gap. This approach can also provide valuable insights regarding the potential timeframe for ASF introduction into new regions.

In the present study, the suitability of a methodological approach to assess the age of the carcass was evaluated based on the molecular detection of β-actin and mitochondrial DNA (mtDNA). 

Since previous studies have shown that the detection of β-actin protein in biological material decreases with aging [14], and that β-actin degradation is positively correlated with the post-mortem interval (PMI) [20], we assumed that this highly conserved protein could serve as a potential indicator for determining the age of wild boar carcasses.

However, as a result of our study, the molecular detection levels of β-actin appear to be characterized by noticeable fluctuations over time. During the 18-month study, we repeatedly observed alternating decreasing Cq values within one month, followed by increasing Cq values at the next sampling time point. Such inconsistency in real-time PCR results was also noted for the individual organs examined, particularly in the kidney, muscle, and skin. Interestingly, in the skin samples, higher β-actin Cq values were observed in those stored outdoors compared to the fridge-preserved samples (during the 1st to 6th months and the 8th month). This difference may be due to the drying of skin tissue, which led to a higher DNA concentration in the final sample and, consequently, more efficient PCR amplification [21]. Nevertheless, despite the irregularities in the molecular detection of this protein, there is a general tendency for β-actin levels, expressed as Cq values in PCR, to decline over time. Notably, the bone marrow PCR results showed a fairly regular downward trend, which could serve as a subject for further research in the future.

Similarly to β-actin, mitochondrial DNA has a wide range of applications in the field of forensics, providing valuable information for human identification based on mitochondrial DNA analysis in isolated populations [22]. For this reason, attempts have been made to determine whether changes in the amount of mitochondrial DNA in the examined organs can be used to assess the age of found animal carcasses. In the final results of our study, we found that, in general, mtDNA values decrease over time when comparing the first and last sampling times throughout the entire experiment. Nevertheless, irregularities in the declining mtDNA values were noticeable, characterized by slight increases at certain points during the study, similar to what was observed with β-actin. The inconsistencies in qPCR results indicate that organs such as the kidneys, muscles, or skin should not be used as target sampling matrices for the intended research purpose. In contrast, the qPCR results obtained for the bone marrow showed the least fluctuations and a regular downward trend, suggesting that this organ may be the most appropriate for our purposes. Considering that most wild boar carcasses found contain bones with bone marrow tissue [23], this observation could prove useful. Nonetheless, further studies should be conducted to confirm and enhance the suitability of the molecular method proposed here.

Although they are completely different molecules, both β-actin DNA and mtDNA share the common feature of undergoing degradation over time [24]. As an isomer of actin, β-actin is a crucial component of cellular microfilaments and is, therefore, present in a wide range of cells and tissues [25]. In contrast, porcine mtDNA is a 16-kbp circular molecule containing a non-coding region and a displacement loop (D-loop), which houses regulatory sequences responsible for controlling mtDNA replication and transcription [26]. By selecting both DNA molecules as qPCR targets in this study, we assumed that their progressive degradation would be reflected in gradually increasing Ct values over time.

Overall, the detection times for both DNA molecules were similar but varied depending on the organ examined. For instance, β-actin DNA was detectable for a longer period in skin and bone marrow compared to mtDNA, while mtDNA had a longer detection period in kidney and muscle, unlike β-actin. This discrepancy may stem from differing initial levels of β-actin and mtDNA in each organ. Additionally, qPCR results for mtDNA exhibited lower standard deviations compared to β-actin when tested in triplicates, suggesting an advantage for mtDNA in terms of result reproducibility. As previously suggested, this may be due to the better performance of primers targeting mtDNA than those used for β-actin detection [13]. Nevertheless, the fact that mtDNA has a higher copy number than β-actin DNA, which results in better DNA quality and more efficient PCR amplification, should not be disregarded [27]. Since a high positive correlation between β-actin and mtDNA contents was found in most of the tested organs, a similar pattern of degradation during corpse decomposition can be expected.

Based on the results of this study, bone marrow can be considered the preferred matrix for assessing PMI. However, it should be noted that these results are indicative and should support other methods of estimating the age of the remains, while also considering the factors discussed below. Proposed PMI expressed as mean Cq values with 95% CI are presented in Table 2.

Recently published studies showed that in the case of ASF-affected animals, the DNA levels may change. While mtDNA levels can increase up to 30-fold in the serum of infected pigs [28], little is known about infection-induced changes in specific organs. It can only be assumed that mtDNA values may also be elevated in sick animals compared to healthy pigs. Similarly, there are few reports on the effect of ASFV infection on host β-actin levels. It has been confirmed that β-actin is involved in various cellular processes, such as facilitating the entry of Bovine Viral Diarrhea Virus (BVDV) into bovine cells [29] and possibly plays a similar role during infection with Classical Swine Fever Virus (CSFV) [30]. If β-actin were involved in ASFV entry into pig cells, one would expect its levels to fluctuate. Therefore, our study results cannot be directly applied to ASFV-infected pigs or wild boars, as the levels of certain molecules may vary during viral infection.

The decomposition of a corpse involves many biological processes that are strongly influenced by temperature, including microbiological activity, scavenger activity (e.g., insects), and oxygen tension [31]. Similarly, our research confirmed that temperature significantly impacts the content of mtDNA and β-actin. More specifically, we found that the Cq values of β-actin and mtDNA were more stable at a constant temperature of approximately 4 °C, and both molecules were generally detectable for a longer duration in fridge-preserved samples. The decomposition process clearly varies with the seasons, progressing more quickly in summer and more slowly in winter [32]. It can be assumed that if the experiment had started at a different time, such as in winter, the results might have differed slightly from those presented here. In such a scenario, the initial environmental temperature would likely be much lower, potentially slowing down the decomposition process while simultaneously maintaining DNA stability within the tested samples [33]. Conversely, during the summer months, the decomposition process accelerates, leading to the rapid inactivation of the virus. Consequently, this may hinder the development of an epidemic [34,35,36].

According to Sutherland’s research [37], body weight influences the rate of decomposition of carcasses, with smaller pig carcasses (up to 20 kg) decomposing more rapidly than larger ones. However, other studies [38] have noted that, during the early stages of decomposition, heavier carcasses decompose more quickly, while body weight does not significantly impact the rate of decomposition in the later stages. In our study, we examined four different organs—kidney, muscle, skin, and femur—harvested from the animal carcasses and placed individually in separate cages. This suggests that individual organs may decompose differently, resulting in varying degradation kinetics for mtDNA and β-actin compared to whole carcasses.

In addition to outdoor temperature, insect activity plays a crucial role in the decomposition processes [39]. Various insects are recognized as significant environmental factors that accelerate the decomposition of carcasses [40], and serve as reliable indicators for estimating the post-mortem interval (PMI) of decomposing remains [24]. Research conducted by Denno and Cothram [41] found that the size of carcasses may correlate with the density of certain insects detected. Scavenger insects are more prevalent in larger carcasses than in smaller ones, further emphasizing the importance of carcass size in determining its age [42]. In our experiments, insects likely had a substantial impact on the biological material tested, contributing to decomposition over time. Starting from the 6th month of the study, we observed significant mass loss in certain organs, particularly in muscle and kidney tissues, attributed to insect activity. Higher outdoor temperatures during the study period, particularly in spring and summer, are known to increase insect activity, which ultimately leads to the rapid degradation of soft tissues [37]. Consequently, we were unable to conduct molecular testing of both parameters in triplicate for all organs, as originally planned.

In summary, both tested parameters—β-actin and mtDNA—exhibited a decreasing trend in their levels over time. Despite noticeable irregularities in the declines across individual organs, a general downward pattern for both β-actin and mtDNA is evident. Notably, the most consistent declines were observed in bone marrow, as discussed above, which could still be collected even in the final stages of carcass decomposition.

Given that determining the exact PMI is often challenging and not always feasible, a more achievable research goal would be to at least approximate it [43]. Although the conducted experiment did not allow for definitive conclusions regarding the reliability of β-actin and mtDNA as parameters for estimating the age of animal carcasses, it provided valuable insights into the degradation dynamics of these molecules in selected tissues. A strong positive correlation was observed between β-actin DNA and mtDNA, indicating a similar tendency for decreasing Cq values, particularly in the bone marrow samples. Further research considering various environmental scenarios is essential to establish precise Cq intervals for approximating the age of Suidae remains. Additionally, the impact of factors such as disease presence, insect activity, and carcass size should be taken into account to create a standardized reference framework.

## 4. Materials and Methods

### 4.1. Samples 

Tissue and organ samples examined in the experiment included kidney, bone marrow (extracted from femur bone), skin, and muscle tissue (*musculus longissimus dorsi*) originating from three (n = 3) commercially obtained pork carcasses (*Sus scrofa domesticus*). Separately for each animal carcass, a set of samples (kidney, femur bone, skin, muscle) was placed in a steel protective cage (protection against wild animals) and located randomly in the forest area. Simultaneously, a corresponding panel of samples taken from the same animal carcasses (approximately 0.1 g) was placed in 2 mL tubes and stored in a refrigerator at 4 °C for the whole duration of this experiment (0–18 months).

### 4.2. Sample Collection 

Individual samples of tissues and organs (triplicate) placed in the forested area (approx. weight 0.1g) were collected into 2ml tubes. Samples stored at 4°C were ready for further processing. The outdoor-kept samples were collected at monthly intervals over a period of 18 months (March 2022–October 2023) as long as the detection of the genetic material, i.e., mitochondrial DNA (mtDNA, Cq value < 35) and β-actin (Cq value < 40) was possible. 

### 4.3. DNA Extraction

Phosphate-buffered saline (PBS) was added to each tube containing the sample to achieve 10% (*w*/*v*) homogenate. Homogenization was performed using TissueLyser II (Qiagen, Hilden, Germany). 200 µL of the obtained 10% homogenates were used as matrices for further DNA extraction. Manual column extraction was carried out using QIAmp DNA Mini Kit (Qiagen, Hilden, Germany) following the manufacturer’s protocol. Additionally, an in-house validated positive control for the extraction of both porcine-origin DNAs was implemented. The DNA samples were stored at −20 °C for further processing immediately after the performed extraction procedure.

### 4.4. PCR Analysis 

Real-time PCR was used for detection of β-actin and mtDNA amounts present in the samples. The detection of porcine-specific DNA was carried out using PrimePCR™ Probe Assay: BETA-ACTIN, Pig ref. No. qSscCEP0032507, Bio-Rad (beta-actin DNA) and DNA SureFood^®^ Animal ID Pork IAAC, ref. no. S6114, R-Biopharm AG (mtDNA) according to the manufacturer’s manual. PCR targeting β-actin DNA and mtDNA were run in two different thermocyclers, i.e., Applied Biosystem 7500 Fast Instrument (ThermoFisher Scientific, Waltham, MA, USA) and LightCycler 480 (Roche, Basel, Switzerland), respectively. For each internal organ within the whole experiment period a single run was performed according to the manufacturers’ protocols. The PCR results were compared with Cq values for the positive controls provided in the PCR kit (mtDNA) or with in-house validated (β-actin DNA).

### 4.5. Temperatures and Humidity Data

Data presented in Figure S1 was shared on the public domain by Institute of Meteorology and Water Management in Poland https://danepubliczne.imgw.pl/.

### 4.6. Statistical Analysis 

The statistical analysis and graphical representation of the analyzed data were performed in GraphPad Prism software (GraphPad 8.4.3., La Jolla, CA, USA). 

## Figures and Tables

**Table 1:** Cq values of β-actin and mtDNA detected in outdoor-stored samples. The data illustrate the duration of DNA detectability and the difference between the initial and final Cq values.

Tissue/Organ	Last Month of Detection		Cq Value in the Last Month of Detection		Min Cq (Mean)		Max Cq (Mean)		Difference Between the First and the Last Measurment ΔCq	
	β-Actin	mtDNA	β-Actin	mtDNA	β-Actin	mtDNA	β-Actin	mtDNA	β-Actin	mtDNA
Bone marrow Sd	13	11	39.22 ^a^	27.96 ^a^	28.31 (±0.84)	18.28 (±1.09)	39.22 ^a^	29.16 (±1.27)	10.91	9.68
Kidney Sd	11	12	35.45 ^a^	29.92 ^a^	22.63 (±0.77)	13.09 (±2.25)	35.45 ^a^	29.92 ^a^	7.57	15.86
Skin Sd	13	9	37.34 (±0.84)	26.65 ^a^	26.52 (±0.77)	18.84 (±0.21)	39.48 (±0.16)	29.17 (±1.94)	10.82	7.81
Muscle Sd	6	7	32.82 (±1.21)	17.64 ^a^	25.29 (±0.15)	16.13 (±0.52)	33.60 (±3.6)	21.95 (±1.33)	3.57	1.16

^a^—Detection in one replicate. Sd—Standard deviation.

**Table 2:** Post-mortem intervals based on mean Cq values with 95% CI. Assessed for bone marrow.

PMI (Months)	Mean Cq Value (95% CI)		Range (Cq)	
	β-Actin	mtDNA	β-Actin	mtDNA
0−3	29.58 (±1.46)	21.42 (±2.09)	25.99−33.95	17.16−25.24
4−6	36.13 (±0.81)	25.26 (±1.73)	33.64−37.35	22.18−28.77
7−11 */7−13 **	38.26 (±0.54)	27.88 (±0.74)	36.99−39.65	26.79−30.95
>11 */>13 **	undetectable	undetectable	undetectable	undetectable

PMI—post mortem interval; *—for mtDNA; **—for β-actin.

## Data Availability

Data available on request.

## Supplementary Materials

The following supporting information can be downloaded at: https://www.mdpi.com/article/10.3390/ijms252111674/s1.
