# Supplementary figures and images for: Evaluation of β-Actin and Mitochondrial DNA Levels in Determining the Age of Suidae Remains
Source: Int J Mol Sci. 2024 Oct 30;25(21):11674. doi: 10.3390/ijms252111674 (PMC11546941; doi:10.3390/ijms252111674)

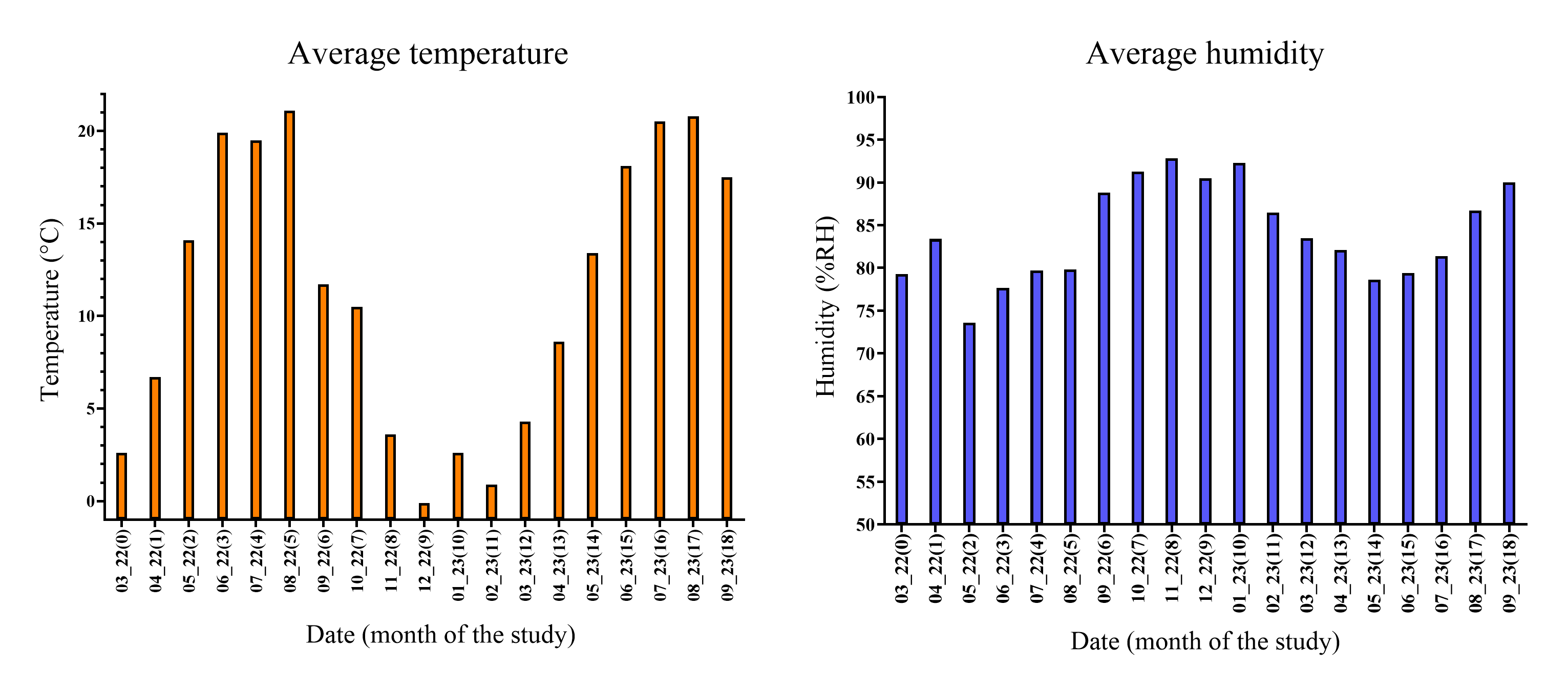

Supplement: Supplementary file 1 [file ijms-25-11674-s001.zip › ijms-3272434-supplementary/Figure S1- Average temperatures and humidity during the study.tif]
